# Principal component analysis revisited: fast multitrait genetic evaluations with smooth convergence

**DOI:** 10.1093/g3journal/jkae228

**Published:** 2024-10-21

**Authors:** Jon Ahlinder, David Hall, Mari Suontama, Mikko J Sillanpää

**Affiliations:** Department of Tree Breeding, Skogforsk, Box 3, Tomterna 1, Sävar SE-91821, Sweden; Department of Tree Breeding, Skogforsk, Box 3, Tomterna 1, Sävar SE-91821, Sweden; Department of Ecology and Environmental Science, Umeå University, Umeå SE-90736, Sweden; Department of Tree Breeding, Skogforsk, Box 3, Tomterna 1, Sävar SE-91821, Sweden; Research Unit of Mathematical Sciences, Oulu University, Oulu FI-90014, Finland

**Keywords:** PCA, Loblolly pine, Scots pine, BLUP, linear mixed-effect model, convergence, genetic correlation, Plant Genetics and Genomics

## Abstract

A cornerstone in breeding and population genetics is the genetic evaluation procedure, needed to make important decisions on population management. Multivariate mixed model analysis, in which many traits are considered jointly, utilizes genetic and environmental correlations between traits to improve the accuracy. However, the number of parameters in the multitrait model grows exponentially with the number of traits which reduces its scalability. Here, we suggest using principal component analysis to reduce the dimensions of the response variables, and then using the computed principal components as separate responses in the genetic evaluation analysis. As principal components are orthogonal to each other so that phenotypic covariance is abscent between principal components, a full multivariate analysis can be approximated by separate univariate analyses instead which should speed up computations considerably. We compared the approach to both traditional multivariate analysis and factor analytic approach in terms of computational requirement and rank lists according to predicted genetic merit on two forest tree datasets with 22 and 27 measured traits, respectively. Obtained rank lists of the top 50 individuals were in good agreement. Interestingly, the required computational time of the approach only took a few seconds without convergence issues, unlike the traditional approach which required considerably more time to run (7 and 10 h, respectively). The factor analytic approach took approximately 5–10 min. Our approach can easily handle missing data and can be used with all available linear mixed effect model softwares as it does not require any specific implementation. The approach can help to mitigate difficulties with multitrait genetic analysis in both breeding and wild populations.

## Introduction

Phenotyping is a critical process in any breeding program with the aim to improve the genetic level of the traits of interest. By accurately characterizing the traits, breeders can make informed decisions about which individuals to select in breeding populations to achieve expected increase in genetic merit shown as increased productivity, quality, vitality depending on the breeding objective. In the near future, many novel high-throughput phenotyping techniques could transform how traits are defined and recorded and could easily reach thousands. For example, remote sensing techniques, such as LiDAR (Light Detection and Ranging), which can capture detailed 3D structural information about crops and trees including canopy height, width and architecture, disease status and condition to name a few (Jin *et al*. 2021). Another high-throughput example involves gene expression data, where linear mixed effect models (LMM) have been used to identify sources of variation in human medicine studies of HIV infection (Yu *et al*. 2019), identifying genotype-by-environment (G×E) interactions in body mass index (Moore *et al*. 2019) and human brain regions (Trabzuni and Thomson 2014). In a plant breeding application, Runcie *et al*. (2021) showed how to use LMM to jointly analyze grain yield and hyperspectral reflectance traits measured in wheat (*Triticum aestivum*) field trials.

Multitrait LMM analysis was introduced in quantitative genetics by Henderson and Quaas (1976) and encompass both genetic covariance component estimation and estimation of breeding values (EBV). Compared to analyzing each trait separately, the advantages of multitrait analysis are:

- increased prediction accuracy of breeding values for un-phenotyped individuals,
- increased statistical power as available data are more efficiently used,
- increased parameter estimation accuracy by exploiting correlations between traits.

In particular, multitrait LMM analysis can provide more accurate estimations in the case of traits with a low heritability (i.e. a proportion of trait variation attributable to genetic factors), populations of small size or if missing data are present (Persson and Andersson 2004; Guo *et al*. 2014). Accurate estimation of variance components and functional parameters, such as heritabilities and genetic correlations, is important because prediction error variances for estimated random effects increase as the differences between estimated and true values of variance components increase (Nishio and Arakawa 2022). Many studies have been published comparing the performance of single and multiple-trait LMMs. For example, Alves *et al*. (2018) compared EBVs obtained from both multitrait and single-trait LMMs via the best linear unbiased predictor (BLUP) for tree height, DBH, and tree volume in *Eucalyptus ssp.* and predicted higher selection response with the multitrait BLUP analysis. Using simulations, Guo *et al*. (2014) showed that for traits with missing data, the EBVs obtained in the multiple-trait analysis resulted in more reliable genomic predictions.

Unfortunately, the number of parameters in multitrait LMMs grows exponentially with the number of traits due to added pair-wise correlation parameters, and the required computational effort therefore grows even more because of the need to invert a (likely) large coefficient matrix at each iteration in the inference procedure, at least for most available algorithms, such as restricted maximum likelihood (REML) (Patterson and Thompson 1971) or Bayesian blocked Gibbs sampling (Waldmann *et al*. 2008). For example, often various convergence problems arises, and this can lead to unstable parameter estimates (Misztal 2008; Johnstone and Titterington 2009). In most practical applications, only a few traits can be analysed simultaneously, which is not optimal as shared information via correlations is not used efficiently in the inferential procedure, causing biased parameter estimates, both for location (i.e. breeding values) and scale (covariance components and heritability). Methods that can circumvent this problem would be sought after.

A number of alternative approaches that tries to circumvent the problems of standard multitrait LMM analysis have been suggested in the literature. Kirkpatrick and Meyer (2004) suggested the use of reducing the rank of the covariance matrix by principal component analysis (PCA) or by factor analytic (FA) models to improve multitrait LMM analysis. By directly estimate the leading principal components (PCs), most of the important information is kept while reducing the computational burden to estimate the covariance matrices (Kirkpatrick and Meyer 2004; Meyer 2007, 2009). This can make the model easier to estimate and interpret, especially in advanced breeding programs where the full covariance structure is complex. Meyer (2007) showed how this approach could be used for selection and multitrait LMM analysis of carcass traits for Angus cattle, where she suggested that the first seven of the PCs were sufficient to obtain estimates of breeding values without loss in the expected accuracy of evaluation. The approach has recently been shown to reduce computational burden in dense genomic marker-derived covariance matrices by Meyer (2023). However, a drawback is that there is an obvious loss of information when the rank of the covariance matrix is reduced, information that could be important for some of the traits included.

The use of dimension reduction techniques to simplify the covariance structure have been a popular choice in crop and forest tree breeding when estimating G×E interactions in multitrait LMM evaluations (Piepho 1997; Smith *et al*. 2001; Burgueño *et al*. 2011; Li *et al*. 2017; Calleja-Rodriguez *et al*. 2019). In a review of G×E in forest tree breeding, Li *et al*. (2017) reviewed analytical methods for inferring G×E effects, including FA modeling, and its application in analysis of field trials of forest tree species including Pine spp, Eucalypt spp, Spruce spp, and Poplar spp. Calleja-Rodriguez *et al*. (2019) incorporated factor analysis to reduce the rank of the covariance matrix which enabled the incorporation of 19 traits simultaneously into the multienvironment LMM analysis of Scots pine (*Pinus sylvestris*) field trials. As a result, they found that the main driver of detected G×E was differences in temperature sum among trial sites. Poupon *et al*. (2023) analyzed the mean annual height increment, the mean annual diameter increment, and wood density in a series of field trials of European larch using FA models: the inferred genetic correlations between sites showed low to high G×E, with growth traits exhibited more G×E than wood density.

Another popular approach in various breeding scenarios is to perform canonical transformation to improve the performance of multitrait LMMs (Itoh and Iwaisaki 1990; Ducrocq and Chapuis 1997; Yang *et al*. 2022), in which a matrix decomposition technique is applied on both genetic and residual covariance matrices. After the transformation is applied, BLUP values can be computed for each trait using univariate LMMs. Then the obtained solution can be back transformed to the original scale, which thereby facilitates interpretation. Unfortunately, a typical requirement for canonical transformation is that covariance matrices either need to be known or ad-hoc estimated before the transformation: this limits the usefulness of the approach as uncertainties in the estimation procedure is not accounted for.

Instead of simplifying the covariance structure, a more direct approach would be to consider transforming the phenotypic traits. The use of PCA to simplify multitrait LMM analysis by operating on the phenotypic trait records is not new and have previously been used to perform genetic variance component and heritability estimation (Atchley and Rutledge 1980; Houle *et al*. 2002). Chase *et al*. (2002) used PCA of skeletal variation in a population of Portuguese water dogs to reveal groups of traits defining skeletal structures and associate it with quantitative trait loci (QTLs). A related PCA-based approach has been proposed for linkage analysis (Ott and Rabinowitz 1999) and genome-wide association analysis (Aschard *et al*. 2014; Zhu *et al*. 2018). The advantages in breeding value and genetic variance component inference of using PCA is that it can handle a large set of traits by transforming them into orthogonal PCs which can be seen as trait combinations with similar characteristics that cannot be measured directly. As each PC is orthogonal, they can be analyzed independently with univariate models. This procedure would be very fast and converge very quickly as opposed to multivariate analysis of a large set of traits, especially when dealing with unbalanced longitudinal data (Adjakossa *et al*. 2016) or with large sets of predictors (Lozano *et al*. 2023). One problem with this approach is that it cannot handle missing data, at least not the standard single value decomposition (SVD) approach, which restrict its use in general breeding applications. Another drawback is that even though the PCs are orthogonal to each other in the phenotypic space, genetic and environmental values are not necessarily uncorrelated between PCs. Thus, by using univariate analyses of the PCs might reduce accuracy of obtained EBVs as compared to when full multivariate analysis of PCs are used. Thus, more effort into using PCA directly on the phenotypic profiles which includes missing data are warranted and to further investigate the effects of nonzero genetic covariances between PCs.

A similar approach using FA modeling, which operates on the response matrix, have recently been proposed (Runcie and Mukherjee 2013; Runcie *et al*. 2021). By introducing latent variables via a mixed effect factor model, all sources of correlation among the traits can be accounted for and corresponding univariate independent LMMs could be analyzed. With this approach, MegaLMM (Runcie *et al*. 2021), three plant breeding data sets with tens-of-thousands of traits were analyzed, and obtained results showed improved prediction accuracy of genetic values and improved computational speed compared to results obtained by traditional methods. As a model-based approach it can handle missing data, but it needs a special software implementation which limits its general use.

Here, we aim at improving (co)variance component and breeding value estimation of large-scale phenotyping efforts by re-introducing PCA dimension reduction technique to obtain reduced space traits. This suggested method can easily be analysed with standard univariate LMMs. In doing so, we circumvent the problems of convergence in the REML analysis to estimate covariance components, at a fraction of the required computational time, compared to a multivariate analysis. A novelty in this proposed approach is how missing data can be handled efficiently in the ordination step by utilizing a model-based PCA for imputation. Two typical forest tree data sets are used to highlight the performance of the approach: one Scots pine field trial included in the north Swedish breeding program and one Loblolly pine breeding population with traits scored in several trials. Because all data were preadjusted for trial-specific design and environmental effects, the continuous nature of the adjusted data facilitated the PCA analysis.

## Materials and methods

### Multitrait LMM

Under Gaussian assumptions, the multivariate version of the LMM was proposed by Henderson and Quaas (1976), and can be written as:


(1) y=Xb+Za+e,

where y is the response vector containing each of *m* traits represented sequentially for *n* individuals in a single column vector (i.e. of size nm×1). This is obtained by taking vec-operation of the multivariate observation matrix of dimension n×m. X is a nm×pm design matrix for fixed effects (with ones, zeros or regression measurements as their elements) in *p* fixed effects in *m* traits. This is a block-matrix with *m* blocks of size p×n. Similarly, b represents the fixed-effects coefficient vector with dimension *pm*, Z is the design matrix for random effects with dimension nm×nm, a denotes the random-effects vector (i.e. polygenic additive genetic effects) with dimension *nm*, e represents the error vector (i.e. residuals) of size *nm*. For a and e, the associated covariance matrices G and R needs to be specified:


(2) $$a=\left(\begin{array}{c} a_{1}\\ a_{2}\\ \vdots \\ a_{m} \end{array}\right),\;var(a)=G=\left(\begin{array}{cccc} G_{11} & G_{12} & \ldots & G_{1m}\\ G_{21} & G_{22} & \ldots & G_{2m}\\ \vdots & \vdots & \ddots & \vdots \\ G_{m1} & G_{m2} & \ldots & G_{mm} \end{array}\right).$$

Now, let $\sigma_{aii}^{2}$ be the genetic variance of trait *i*, and $\sigma_{aij}$ is the genetic covariance between two traits *i* and *j* within an individual, the genetic covariance matrix $G_{0}$ can be defined as:


(3) $$G_{0}=\left(\begin{array}{cccc} \sigma_{a11}^{2} & \sigma_{a12} & \ldots & \sigma_{a1m}\\ \sigma_{a21} & \sigma_{a22}^{2} & \ldots & \sigma_{a2m}\\ \vdots & \vdots & \ddots & \vdots \\ \sigma_{am1} & \sigma_{am2}^{2} & \ldots & \sigma_{amm}^{2} \end{array}\right).$$

For the residuals, the covariance matrix R has a similar definition as G, but uses the identity matrix (assuming homoscedasticity) in place of the additive genetic relationship matrix. Furthermore, the random effects are assumed to follow a multivariate normal distribution $a∼MVN(0,G_{0}⊗A\sigma_{a}^{2})$, where $G_{0}$ is a m×m genetic covariance matrix, A is a n×n additive genetic relationship matrix, $\sigma_{a}^{2}$ is the additive genetic variance component, and ⊗ is the Kronecker product. The residuals are assumed to be multivariate normally distributed as $e∼MVN(0,R_{0}⊗I\sigma_{e}^{2})$, where $R_{0}$ is a m×m  *within individual* residual covariance matrix and I is an n×n identity matrix, viz., *between individual* residual covariance matrix and $\sigma_{e}^{2}$ is the residual variance component.

### Standard singular value decomposition-based PCA

PCA reduces the dimensionality of data while preserving its essential information (Hotelling 1933; Wold *et al*. 1987; Jolliffe 2002). PCA is computed for n×m multivariate observation matrix Y, where *n* is the number of individuals and *m* traits. If n≤m, it is practical to calculate it for n×n matrix of $YY^{\prime }$. Otherwise, it is calculated for m×m matrix of $Y^{\prime }Y$. Let us represent a scaled symmetric covariance matrix $YY^{\prime }$ as a product of two orthonormal matrices Q containing orthogonal unit vectors as columns and one diagonal matrix D such as ${YY}^{\prime }={QDQ}^{\prime }$. Orthogonality of Q means that $Q^{\prime }Q=I$. D is the square diagonal matrix with the singular values of Y on the diagonal. Now, if we multiply both sides from left and right with $Q^{\prime }$ and Q, respectively, we obtain $Q^{\prime }YY^{\prime }Q=D=diag(\lambda_{1},\lambda_{2},\ldots ,\lambda_{p})$, where the right hand side of equation contains eigenvalues of matrix $YY^{\prime }$ in the diagonal in the ascending order $\lambda_{1}\geq \lambda_{2}\geq \ldots \geq \lambda_{p}$. This decomposition is related to singular value decomposition (SVD) (Golub and Van Loan 2013). For PCA, the singular values are the square roots of the eigenvalues of the covariance matrix, and both eigenvalues and singular values provide insights into the phenotypic variability and importance of different components (eigenvectors or basis vectors) in transforming and summarizing the observed phenotypic profiles.

### Model-based PCA

PCA is a linear transformation of the covariance matrix of the data to the space where different directions are independent. As an alternative to the algorithmic-based exact PCA is to fit the transformation model to the data statistically using a probabilistic model-based approach (i.e. observed data points are generated from a probabilistic distribution) (Tipping and Bishop 2002). This of course requires distributional model assumptions which increase transparency but makes the transformation somewhat noisy. One advantage is the possibility to include handling of missing values as part of the hierarchical model. Oba *et al*. (2003) suggested a Bayesian model-based version of PCA, or BPCA, which simultaneously fits a probabilistic model and infer latent variables (i.e. PCs). The main step that include the missing value imputation in BPCA is the PC regression step: for the i:th trait $y_{i}=\sum_{l=1}^{\;p}x_{l}w_{l}+\epsilon$ where $w_{l}$ is the l:th principal axis vector and $x_{l}$ is the linear coefficient to be estimated (also called the factor score), *p* is the total number of components used, and ϵ is the residual. The goal is to minimize the sum of squared errors $\|\epsilon {\|}^{2}$ for Y (i.e. for all traits) by using PCA. As we have missing data, the principal axis matrix W can be divided into a complete and missing data part: $W=(W^{obs},W^{miss})$. The factor scores x are then obtained by minimizing the residual error for the observed data $y^{obs}$, and then used to obtain $y^{\mathrm{miss}}=W^{\mathrm{miss}}x$. Oba *et al*. (2003) used Bayesian inference, via a variational Bayes algorithm (Bishop 2006) to estimate model parameters and missing records. Interested readers are invited to see Oba *et al*. (2003) for further details of the missing data imputation steps.

### Implementation details

Standard SVD-based PCA were performed using the prcomp function from the R package stats (R Core Team 2022) with default settings. We used the R package pcaMethods (Stacklies *et al*. 2007) to apply BPCA to impute missing data and perform ordination by using the pca function with parameters maxSteps=10,000 and threshold=1e−06. In addition, we also used the missForest R package (Stekhoven and Buehlmann 2012) as a comparison of the effect of missing data imputation. To compare ordinations (i.e. loadings) on different imputed data in the Loblolly pine example, Eucledian distance of the loading matrices were first calculated (dist function) and then compared using a Mantel test (mantel function) available in the Vegan R package (Oksanen *et al*. 2022). Contribution bar plots were created using the R package factoextra (Kassambara and Mundt 2020).

ASReml-R (Butler *et al*. 2023) was used to infer genetic parameters both in the standard bivariate approach and in the univariate analysis of PCs, with the workspace parameter increased to 4,096 mb, and the ai.sing parameter set to true (which tells ASReml-R to continue the fitting process even if it encounters singularities). The inverse of the additive genetic relationship matrix was calculated using the ainverse function in the ASReml-R package.

To infer the association between selection index and PCs, we back-transformed obtained EBVs for the PCs to the original phenotypic scale by ${\hat{G}}_{\mathrm{orig}}={\hat{G}}_{EBV}\Lambda^{\prime }+\mu_{\mathrm{orig}}$, where ${\hat{G}}_{EBV}$ is the obtained EBV for each PC (column) for each individual (row) of size n×p, Λ is the rotation matrix of eigenvectors of dimension m×p, $\mu_{\mathrm{orig}}$ is the original trait mean vector of length *m*. Note that if p=m, i.e. there is no dimension reduction performed, the back-transformation is exact and no information is lost. Rank lists were compared using association test between paired samples with Kendall’s *τ* method implemented in the cor.test function. The null-hypothesis tested was if the true tau was equal to 0 (i.e. no association).

All LMM software’s tested on the first three PCs of the Loblolly pine data were used with default settings. As alternative to the PCA approach, we used factor analysis via the MegaLMM implementation (Runcie *et al*. 2021) with five chains totaling 1,500 iterations and 4 chains sampling from the obtained stationary sampling distributions collecting 125 posterior draws per chain totaling 500 points. The number of latent variables used was set to 10. MegaLMM required the additive genetic relationship matrix which was calculated using the nadiv R package (Wolak 2012).

To perform clustering analysis of the loadings, Ward’s method was used in conjunction with Euclidean distance via the hclust function in R, stats package. All figures were produced using the ggplot2 R package (Wickham 2016).

To calculate standard deviation of the narrow-sense heritability based on estimated variance components and their standard deviations (StdDev), we made use of the following Taylor’s approximation: $\text{StdDev}(C)=\frac{A}{B}\cdot \sqrt{(\frac{\text{StdDev}(A)}{A}{)}^{2}+(\frac{\text{StdDev}(B)}{B}{)}^{2}}$ assuming absence of co-variation between *A* and *B*, where in our case $A={\hat{\sigma }}_{A}^{2}$, and $B=({\hat{\sigma }}_{A}^{2}+{\hat{\sigma }}_{E}^{2})$. All StdDevs were estimated in respective software bar the rrBLUP package which required additional subsampling.

For further details including R code and data, please visit https://github.com/jonhar97/Reduced_phenotype_MME.

### Analysed Scots pine data

The Scots pine (*Pinus sylvestris L.*) field trial was designed to test the performance of available genotypes in seed orchard 412 Domsjöänget. The trial was established in 1971, located in Vindeln, Sweden $64.18^{\circ }\;\text{N},\;19.34^{\circ }\;\text{E}$ and consisted of 206 full-sib families obtained from controlled crosses of 52 seed orchard parents and five local stand seed sources, totaling 8,160 plants at 3.95 hectare of land. The plants where spaced at 2.2×2.2 meter squares in single tree plots. The trees were measured after 10, 14, 26, and 47 growing seasons for production and quality related traits (Table 1, Supplementary Fig. 1). The trial was thinned after the 26 year measurement. Previous studies have reported moderate heritability estimates for tree height and diameter (Ericsson 1999; Finley *et al*. 2008; Hallander and Waldmann 2009). We used two alternative selection indices with equal weight to all included traits at age 26 (i.e. close to final evaluation of the trial in north of Sweden):

- production using height (Hjd_26) and diameter (Dia_26)
- production and tree stem quality using equal weights for height (Hjd_26), diameter (Dia_26), branch angle (Gvin_26), and (negative) branch diameter (Gdia_26)

To preadjust phenotypic records, we followed e.g. Calleja-Rodriguez *et al*. (2019) by using the following set of predictors:

- fixed effect: intercept,
- random effects: plot, rows within plot, columns within plot,
- residual covariance structure: AR1 autocorrelation term on rows and columns.

**Table 1.** Traits measured in the Scots pine progeny trial.

Trait	Age	Trait type	Number of observations	${\hat{h}}_{1,685}^{2}$	${\hat{h}}_{6,044}^{2}$	Additional info
Dia_14	14	Production	2,765	0.077 (0.035)		Diameter at breast height
Dia_26	26	Production	5,244	0.120 (0.041)	0.147 (0.032)	Diameter at breast height
Dia_47	47	Production	4,302	0.242 (0.055)	0.208 (0.040)	Diameter at breast height
Ftop_47	47	Quality	4,425	0.047 (0.028)	0.041 (0.017)	Number of top shoots
Gant_14	14	Quality	2,767	0.236 (0.055)		Number of branches per whorl at age 14: The sum of the whorls closest below and above 130 cm.
Gdia_26	26	Quality	5,313	0.372 (0.067)	0.320 (0.052)	Average branch diameter compared to surrounding trees
Gdiagr130_14	14	Quality	2151	0.131 (0.045)		The diameter of the largest branch in the branch whorl closest to 130 cm above ground
Gvin_26	26	Quality	5,313	0.382 (0.066)	0.347 (0.052)	Branch angle compared to surrounding trees
Gvingr130_14	14	Quality	2,148	0.479 (0.071)		The angle of the largest branch in the branch whorl closest to 130 cm above ground
Hjd_10	10	Production	6,027	0.112 (0.040)	0.191 (0.037)	Total tree height
Hjd_14	14	Production	5,506	0.262 (0.057)	0.249 (0.043)	Total tree height
Hjd_26	26	Production	5,248	0.573 (0.072)	0.369 (0.055)	Total tree height
Hjd_47	47	Production	4,023	0.390 (0.066)	0.323 (0.052)	Total tree height
Sprant_14	14	Quality	2,898	0.084 (0.035)		Top shoot count
Sprant_26	26	Quality	5,316	0.110 (0.040)	0.147 (0.032)	Top shoot count

SEs are within parenthesis. Estimates of narrow-sense heritabilities for the 1,685 and 6,044 tree subsets are denoted as ${\hat{h}}_{1,685}^{2}$ and ${\hat{h}}_{6,044}^{2}$, respectively.

### Analysed Loblolly pine data

The loblolly pine (*Pinus taeda L.*) breeding population dataset was published by Resende *et al*. (2012), which originated from controlled crosses of 32 parents (22 field- selected F0 plus trees and 10 selected F1 progeny) representing a wide range of accessions from the southeastern USA. A subset of 926 genotypes of the F2 offspring was selected for extensive phenotyping in three replicated studies for growth, developmental, and disease-resistance traits measured at 1, 2, 3, 4 and 6 years Table 2. We defined selection index inspired by Isik and McKeand (2019):

- production index with equal weights on EBVs for height and diameter at age 6
- production and disease susceptibility index with EBVs for height, diameter, and (negative) rust infection at equal weight
- production and wood quality with EBVs for height, diameter, stiffness, and density at equal weight

No economic data were used in the calculation of selection indices. See e.g. Cumbie *et al*. (2012), Hayatgheibi *et al*. (2017) and Fundova *et al*. (2018) for further details about construction of economic weights in index selection for various pine species.

**Table 2.** Traits measured in the Lololly pine breeding population of 861 genotypes.

Test	Age	Trait	Trait type	${\hat{h}}^{2}$	Additional info
Canker	1	LesionUF	Quality	–	Removed because of high precentage missing data
Nassau	1	HT	Production	0.337 (0.093)	
Nassau	2	CWAC	Production		Crown width across the planting beds
Nassau	2	CWAL	Production	0.544 (0.118)	Crown width along the planting beds
Nassau	2	HT	Production	0.646 (0.125)	
Nassau	3	DBH	Production	0.589 (0.123)	
Nassau	3	HT	Production	0.570 (0.121)	
Nassau	4	DBH	Production	0.538 (0.118)	
Nassau	4	HTLC	Production	0.424 (0.102)	Total height to the base of the live crown
Nassau	6	BA	Quality	0.510 (0.111)	Branch angle average
Nassau	6	BD	Quality	0.223 (0.072)	Average branch diameter
Nassau	6	BLC	Production	0.546 (0.118)	Basal height of the live crown
Nassau	6	CWAC	Production	0.553 (0.118)	Crown width across the planting beds
Nassau	6	CWAL	Production	0.409 (0.102)	Crown width along the planting beds
Nassau	6	DBH	Production	0.558 (0.120)	
Nassau	6	HT	Production	0.456 (0.111)	
Rootnum	10	Rootnum	Production	0.087(0.041)	Root number
Rootnum	10	Rootnumbin	Production	0.269(0.080)	Presence or absence of roots
Rust	1	Gall_vol	Disease susceptibility	0.116(0.047)	Susceptibility was assessed as gall volume
Rust	1	Length	Disease susceptibility	0.157 (0.057)	
Rust	1	Rustbin	Disease susceptibility	0.190(0.064)	Presence or absence of rust
Rust	1	Width	Disease susceptibility	0.177(0.061)	
Woodall	4	C5C6	Quality	0.190 (0.066)	5- and 6-carbon sugar content
Woodall	4	Density	Quality	0.114(0.048)	Wood-specific gravity
Woodall	4	LateWood	Quality	0.165 (0.060)	Latewood percentage at year 4
Woodall	4	Lignin	Quality	0.087 (0.041)	Lignin content
Woodall	5	StiffnessTree	Quality	0.318 (0.087)	

SEs are within parenthesis. Estimates of narrow-sense heritability for each trait is denoted ${\hat{h}}^{2}$.

## Results

### PCA-based multitrait LMM analysis of a Scots pine field progeny trial

#### Accurate EBV ranking

Two subsets were extracted from the original data of 8,100 trees to check the robustness and performance of the PCA-based method: one smaller subset of 1,685 tress scored for 15 traits without missing data, and a larger subset of 6,044 trees measured with 10 traits with 13.3 % of missing data (Supplementary Fig. 1, Table 1). Prior to the analysis, all data were spatially adjusted using an first order spatial autoregressive (AR1) model as suggested by Dutkowski *et al*. (2002) to remove micro-environmental variation. First, we focus on the analysis of the complete 15 trait subset to compare the approaches.

**Fig. 1.**
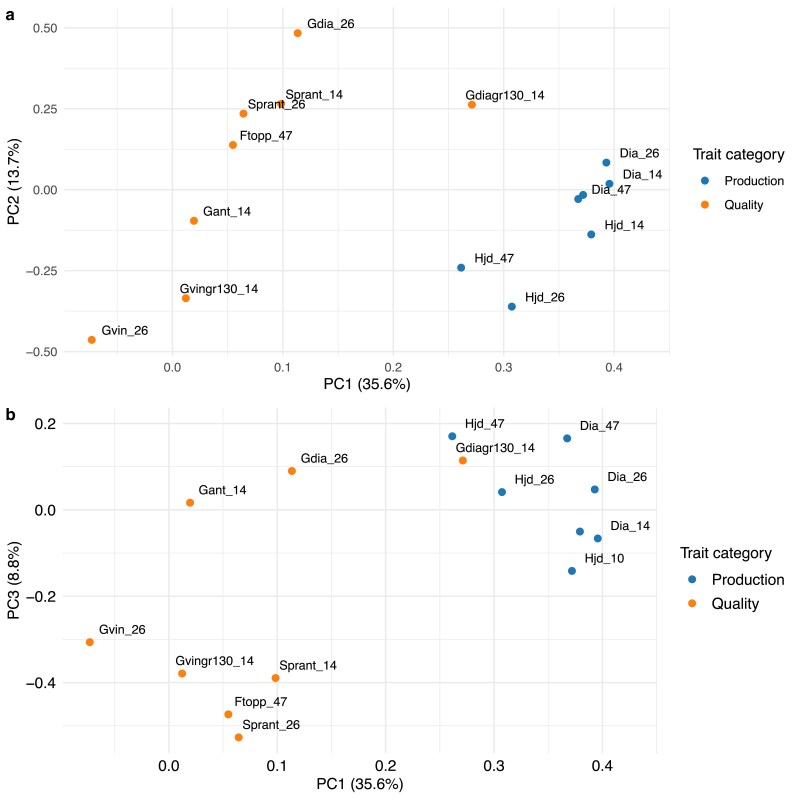
PCA on the 1,685 tree subset with 15 traits measured: a), The loadings of PC one and two with trait categories colored, b) loadings of PC one and three.

**Fig. 2.**
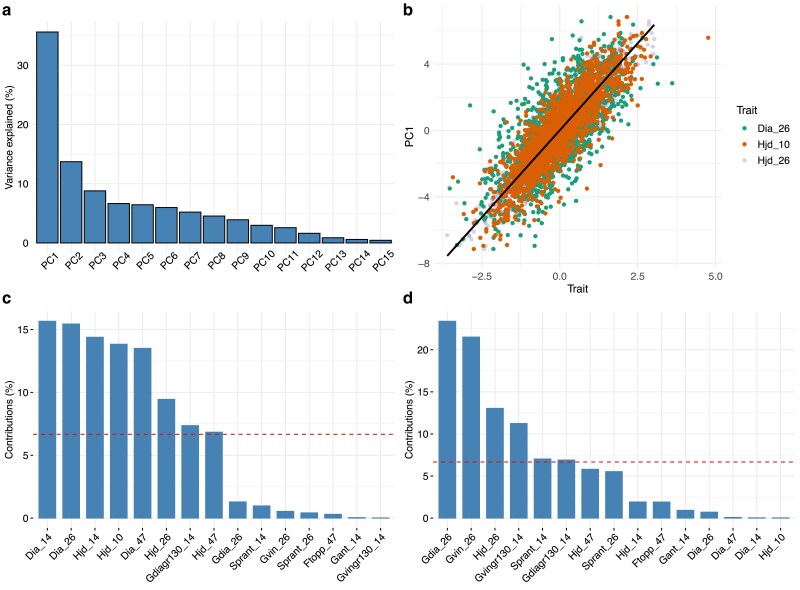
PCA on the 1,685 tree subset with 15 traits measured: a) proportion of phenotypic variation explained by each of the 15 first PCs, b) scatter plot of PC1 scores and highest correlated trait values (Dia_26, Hjd_10, and Hjd_26), c) the contribution of the original traits to phenotypic variation in PC1, and d) the contribution of the original traits to phenotypic variation in PC2.

The PCA revealed strong clustering tendencies among the recorded traits, where the first PC differentiated between production and outer tree quality traits, while retaining a large chunk of the total phenotypic variation (Figs. 1–2). In total, 15 PCs were used to explain all phenotypic variation, where the first three components explained 35.6, 13.7 and 8.8 % of the variation (cumulative: 58.1 %). This manifested as a strong linear correlation between the first component and multiple production traits such as height and diameter at different ages (Fig. 2). To confirm the grouping of the traits using the obtained loadings in the PCA, we performed hierarchical clustering of the loadings which showed the same partition of traits into production and quality groups divided at the highest hierarchy level (Supplementary Fig. 2).

**Fig. 3.**
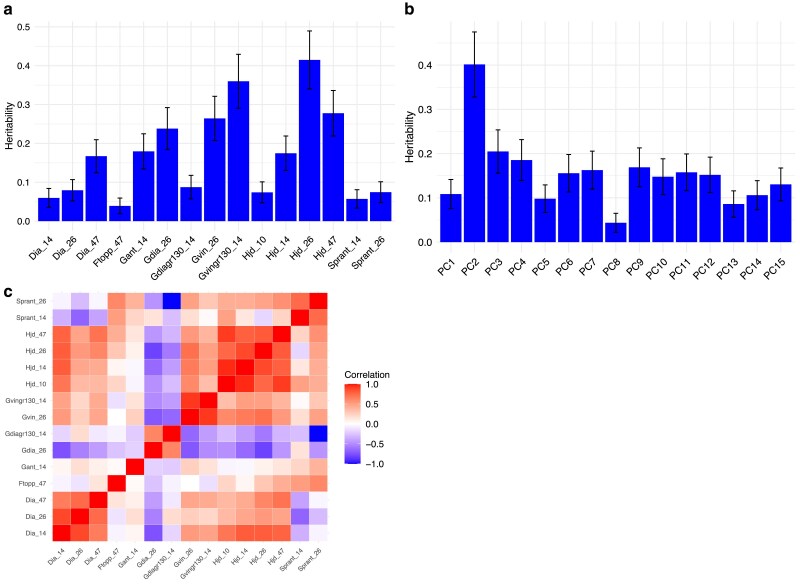
REML pairwise trait analysis on original traits and on PCA transformed traits (i.e. PCs) showing: a) ${\hat{h}}^{2}$ of the original 15 traits using both the bivariate and univariate approaches with SE displayed as vertical lines, b) shows obtained ${\hat{h}}^{2}$ of the 15 PCs analysis, and c) shows the pair-wise estimated correlations obtained with REML between all 15 traits.

The REML analysis on the original traits was carried out in several steps to make the model converge: a) a univariate REML analysis to estimate good starting values of the variance components, b) pairwise bivariate REML analysis of all trait combinations to obtain estimates of genetic and residual covariances between traits (i.e. to fill entries in the $R_{0}$ and $G_{0}$ matrices, and c) a full multitrait mixed model analysis with fixed scale parameters via $G_{0}$ and $R_{0}$ matrices obtained in step b). Thus we were using the fixed parameters to estimate breeding values for all traits simultaneously. The estimated correlations and narrow-sense heritability (${\hat{h}}^{2}$) for all traits is shown in Fig. 3 for both REML analyses of original and transformed traits. The range for ${\hat{h}}^{2}$ the original traits varied from 0.04 (Multiple stems year 47) to 0.57 (Height year 26) (Table 1). The computational time required for these steps where a) 2.51 (0.13) seconds, b) 94.3 seconds (individual runs ranging from 0.17 to 2.57 seconds, mean 0.89 (0.61) seconds), c) the model did not converge after 10,000 iterations (i.e. the log-likelihood maximum was not reached), although we deemed the model found a near optimal solution as the difference in the log-likelihood did not change in its third decimal across 10 iterations: as each iteration took 2.5–3.9 seconds, the total required time in the c) step was 7 h, and 7 min.

Then, univariate REML analyses of all 15 PCs as response variables were performed as comparison, and in all cases converged after 10 iterations in less than a second per PC. Obtained ${\hat{h}}^{2}$ for all PCs are shown in Fig. 3b, and ranged between ${\hat{h}}_{PC8}^{2}=0.061(0.032)$ and ${\hat{h}}_{PC2}^{2}=0.564(0.072)$, with SE within parenthesis. As comparison, the MegaLMM analysis took on average 7 min on the same dataset.

**Fig. 4.**
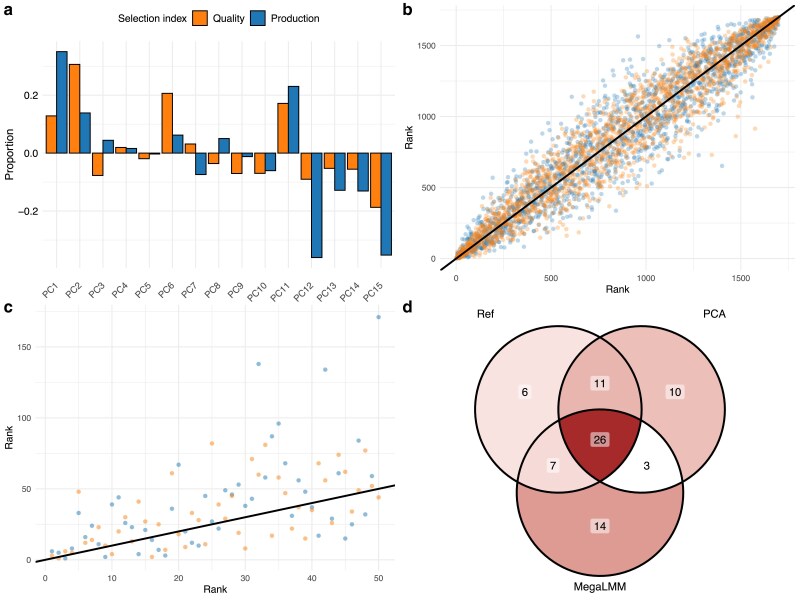
Comparison of tree rank based on estimated breeding values (EBVs) for PCA and standard approaches: a) The contribution of individual PCs to the selection index corresponding to a traditional model both for quality and production-based indices, b) difference in rank of individuals where the rank of EBV based on analysis of original trait is the reference (*x*-axis) and the corresponding rank based on analysis of PC score traits (*y*-axis) for two selection index, c) the same rank differences, but zoomed in on the first 50 reference ranked individuals, and the color corresponds to the two selection indices. Panel d) shows the number of common genotypes among top 50 ranked trees of the production index.

**Fig. 5.**
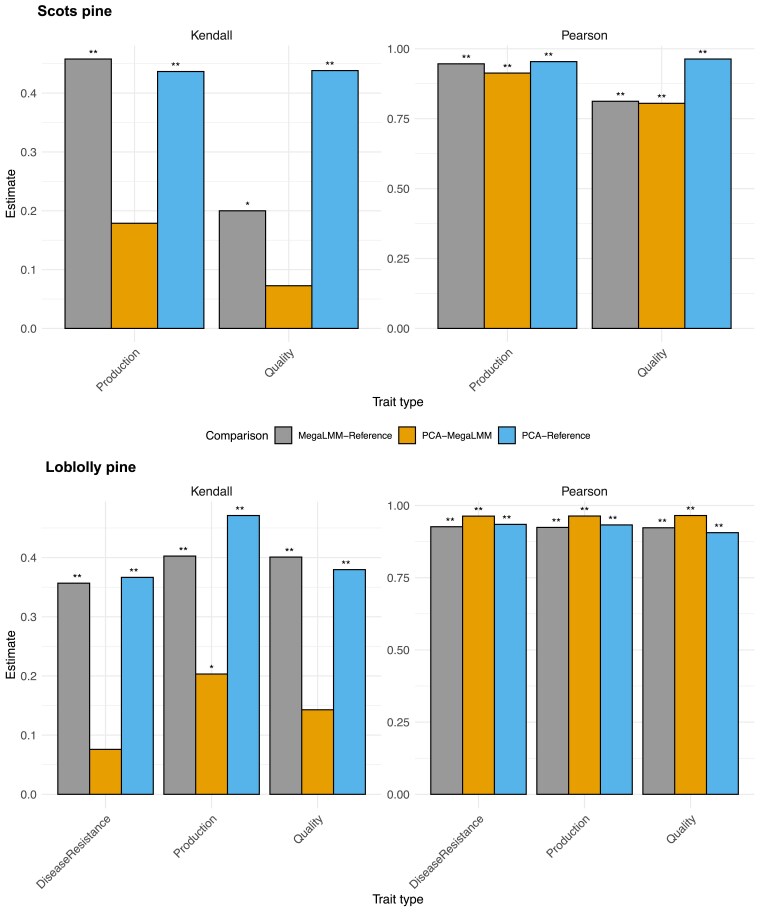
Comparison of calculated correlations obtained for analyses with MegaLMM, PCA, and traditional multivariate approaches. Top row shows the result on the Scots pine data example and the bottom row shows the result of the Loblolly pine case study. Left column shows Kendall’s *τ* based on top 50 rank of indices and right column highlights Pearson product moment estimator based on index of the entire population. Obtained test significance is highlighted as stars on top of the corresponding bar for *P<0.05 and **P<0.01.

From a breeder’s perspective, the most crucial information is the ranking of EBVs and how one can utilize this information to perform selection and crossing (or mating) decisions. We defined two selection criteria: one production based with 50% EBVs for height and diameter at breast height at age 26, and one outer tree quality based with 50% production, 25% branch angle and negative 25% branch diameter, all measured at age 26. As a common breeding objective goal of forest trees is to increase the productivity, we included both height and diameter in both indices. In addition, outer tree quality traits, such as branch angle and branch diameter, will impact wood quality and their improvement are also important long term breeding goals. To make index based on PC traits comparable, we back-transformed EBVs of the PCs to EBVs on the original trait scale. Then we computed two indices (Fig. 4a), where most PCs contributed bar PCs 4 and 5 which contributed very little to both indices, ensuring that all genetic variation was kept in the index, minimizing information loss. Correlations between PCA derived indices and traditional indices was highly significant: r=0.954P<0.0001 and r=0.963P<0.0001, for production and quality indices of all 861 selection candidates (Figs. 4b and 5). In all, the correlation of obtained rank of the top 50 individuals based on selection index values from standard and PCA analysis were positive and significant (Figs. 4c and 5): Kendall’s τ=0.438,P<0.0001, and τ=0.437,P<0.0001, for production and quality index, respectively. Furthermore, correlations of the obtained selection indices of the PCA approach to the reference multivariate method agreed closely to those obtained by MegaLMM (Fig. 5 top panel), while the top ranked part were more similar between PCA and reference than between MegaLMM and reference. This result is supported by the number of overlapping individuals in the top 50 rank lists where PCA and reference-based ranking shared 11 and 16 genotypes for production and quality index respectively, which was not present among the top 50 genotypes obtained by MegaLMM (Fig. 4d, Supplementary Fig. 3). Thus, although not identical, the rank lists resembled each other well in the top 50 rank, and similar response to selection is expected, at a fraction of the required computing effort.

Even though obtained PCs are orthogonal at the phenotypic level, it is not necessary true at genetic and residual levels. To address this, we performed a full multivariate analysis of all PCs to investigate the correlation pattern using both ASReml and MegaLMM. In the REML analysis, logL coverged but not the covariance components, suggesting difficulties in the inference procedure to obtain reliable estimates, with estimates varying considerably for both genetic and residual correlations. In the Bayesian FA model analysis, obtained estimates were around zero for all combinations of PCs (results not shown). Thus, covariance estimates conflicted between methods, but this issue seem to have little influence in practice regarding the rank lists in this example.

#### Missing data can be efficiently handled

Standard SVD-based PCA (SVD-PCA) cannot handle missing data. As data collected from realistic field trials would typically contain at least some proportion of missing observations, in particular if many traits have been measured. To check the effect of missing data on PCA-based multitrait selection, we selected a subset of 6,044 trees scored for ten traits. The data contained 13.3% missing data in total ranging from 17 to 2021 missing observations for tree height at age 10 and age 47, respectively. The Bayesian PCA method (BPCA) was used to impute missing observations with nine PCs explaining 98.3% of the total variation.

To rule out that the imputed data had any impact on the genetic parameter estimates obtained by the REML method, we analyzed the data from 6,044 individuals with 10 traits using the pairwise bivariate REML approach described earlier. We focus on the trait with the largest proportion of missing data (33.4%), tree height at age 47, as the worst-case scenario. The average difference in correlations to all other nine traits was 0.040 (0.035), with standard deviation within brackets. Correlations with some of the traits were overestimated with the imputed data, such as multiple stems and diameter at age 47, with 0.094 and 0.081, respectively. Obtained narrow-sense heritability estimates for the trait were $h_{\mathrm{imputed}}^{2}=0.323(0.052)$ and $h_{\mathrm{NA}}^{2}=0.264(0.044)$. However, this difference had little impact on EBV and ranking of trees as $r_{\mathrm{Imp},\mathrm{NA}}=1.0,P=2.2\times 10^{-16}$, probably because information is shared across correlated traits with much less fraction of missing data.

Taken together, we used imputation via BPCA to keep as much information as possible and there were only slight differences between the BPCA and SVD–PCA approaches on the Scots pine progeny trial data (results not shown). In practice, both methods could be used interchangeably without changing the rank of trees. The need for imputing missing observations might be a bigger concern when it comes to estimating scale parameters but seems to be less important when considering rank of individuals based on EBV.

### PCA allows for rapid multitrait analysis in a *Pinus taeda* pedigree

**Fig. 6.**
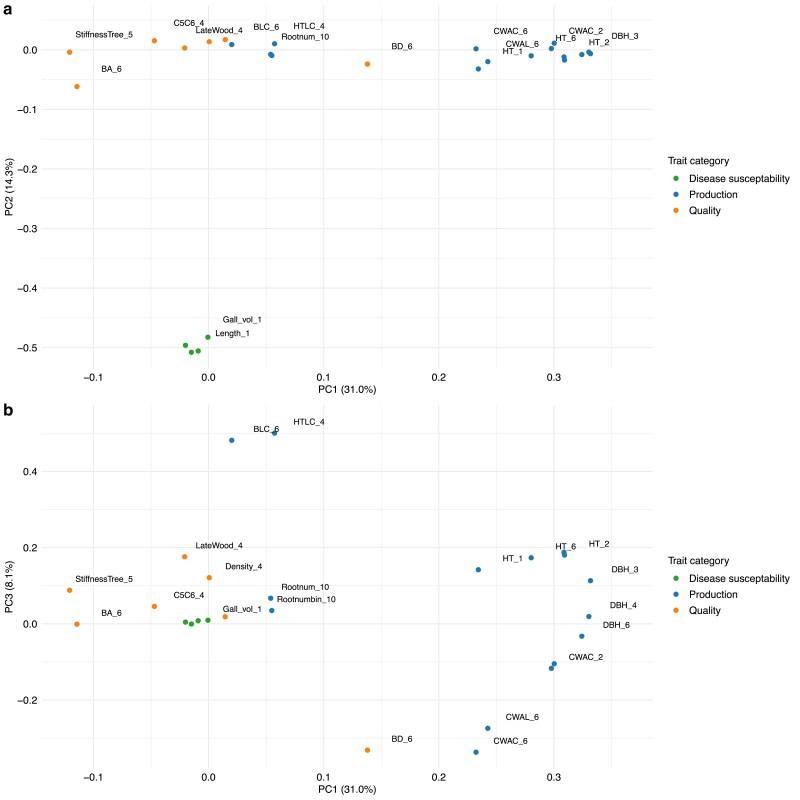
Multitrait LMM analysis on the 26 selected traits in the Loblolly pine dataset. a) Clustering of traits via PCs one and two colored with their respective trait category. The number on the axis labels corresponds to the percentage of phenotypic variation explained. b) PC one and three.

In total, 27 traits were recorded for 926 individual genotypes in the South eastern USA breeding program of Loblolly pine (Table 2). As missing data were present, we first removed traits with >40% missing data, which resulted in the removal of the trait LesionUF_1 (i.e. damage to the tree that is caused by an unidentified factor after one growth season). In addition, individuals with >25% missing data were removed, resulting in 861 pedigree member available for the multitrait analysis. Missing data were imputed using the missForest method. SVD-PCA analysis of all 26 PCs was performed explaining 100% of the total phenotypic variation. The PCA analysis of the 26 traits revealed clustering tendencies of the loadings for similar trait types (Fig. 6a and b), with the first PC explaining 31.4% of the phenotypic variation, while the second and third explained 14.3% and 8.1% respectively. The first PC separates the production traits from the tree quality and disease susceptibility traits, albeit with some of the traits mixed (i.e. at PC1 values close to zero), such as branch diameter year 6 (BD_6), and the total tree height to the base of the live crown (HTLC_4). The second PC clearly separates production and tree quality traits from the disease susceptibility traits. The third PC separated crown width traits and branch diameter with various tree height traits.

To test the impact of missing data imputation method, we also used the BPCA method and simple trait means to complete the data set and performed standard SVD-PCA (Supplementary Fig. 4). The BPCA explained 97.1% using 20 PCs with the first PC explaining 31.8%, while the missForest explained 98.7% in the first 20 PCs while the first PC explained 31.0%. When comparing the scores for the first 20 PCs in both BPCA and missForest imputed data resulted in highly correlated ordinations (Mantel’s r=0.545,P=0.001). Using trait means as imputation resulted in a very similar ordination as the missForest imputation SVD-PCA (results not shown). Thus, the method of imputation had relatively small impact on the resulting ordinations, although using the BPCA seemed to be advantageous if the first PC is of main interest (i.e. production traits).

To examine downstream results (i.e. rank lists) of PCA and multivariate LMM approaches, we followed the same procedure as with the Scots pine example. First, to obtain the starting values of the bivariate REML analyses for estimating variance components, 26 univariate REML analyses were performed. In most cases, the model converged after 4–7 iterations, but in some cases, however, resulted in Log-likelihood not converging, and that some components changed by more than 1% on the last iteration. Each run was very quick, less than a second for all 26 traits. In total, 325 bivariate REML analyses were required to cover all trait combinations to estimate the trait covariance matrix. These analyses took 62 seconds in total, with very mixed convergence statistics ranging from 4 to 704 iterations. Finally, the full multivariate analysis lasted for 12 h 22 min to run 3,000 iterations until convergence, where each iteration took between 11 and 18 seconds. As comparison, univariate PCA LMM took less than a second for each PC and converged after a few iterations. To examine the genetic and residual correlations between the first 10 PCs a full multivariate model analysis were performed with all obtained covariances (i.e. both genetic and residual) was 0, suggesting a total absence of correlations. This finding was confirmed by the MegaLMM analysis of all PCs. MegaLMM analysis of the 26 original traits took 5.3 min on average over 10 repetitions.

**Fig. 7.**
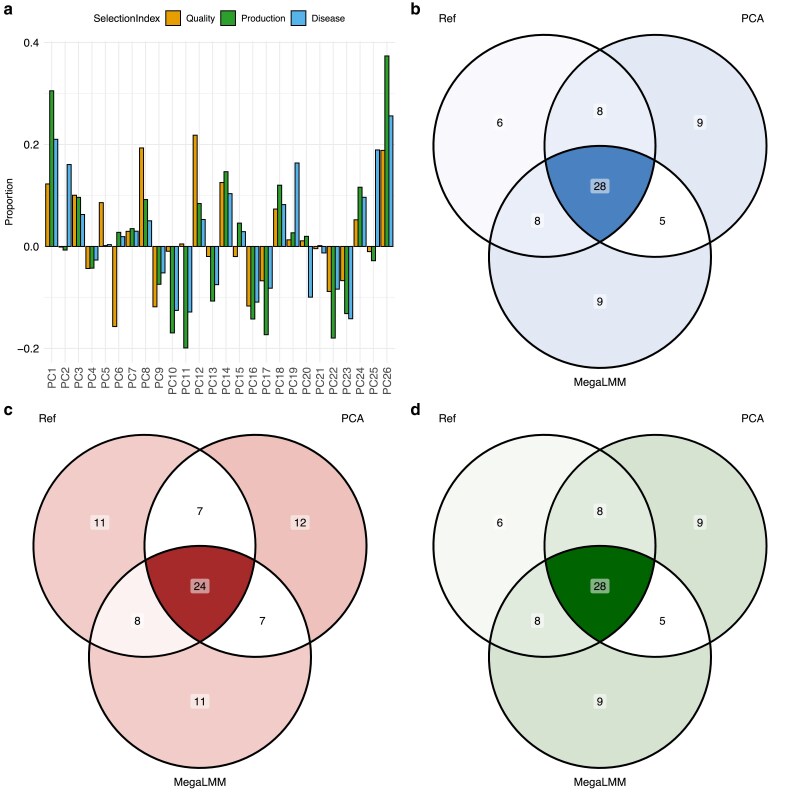
Multitrait LMM analysis on the 26 selected traits in the Loblolly pine dataset: a) Selected PCs for the three different selection indices and their respective contributing proportion to the index. b) common genotypes among top 50 ranked trees for quality index, c) common genotypes among top 50 ranked trees for production index, and d) common genotypes among top 50 ranked trees for disease index.

To compare rank lists, we created three selection indices: one production-based index with 50% height and 50% diameter EBVs, one tree quality index combining height and diameter with tree density and wood stiffness all weighted equally, and finally a disease susceptibility index with height, diameter, and fusiform rust presence. We used back-transformation to combine the PCs which best mimics these indices (Fig. 7a), where all 26 PCs contributed to the indices implying that all available phenotypic variation was utílized. Obtained EBVs of the indices with univariate PCAs LMM analysis were highly correlated to the EBVs of the full multivariate analysis for production index, r=0.933,P<0.0001, for quality index, r=0.906P<0.0001, and for disease resistance index, r=0.935P<0.0001. Unsuprisingly, the rank lists of the top 50 trees obtained with PCA and traditional approach resembled each other for all three selection indices considered (Fig. 5 bottom panels, Fig. 7b): production index, Kendalls τ=0.471,P<0.0001, quality index, Kendalls τ=0.380,P=0.0001, and disease susceptibility index, Kendalls τ=0.367,P=0.0002. These results suggest that similar rank lists can be obtained with the PCA approach for three different indices but at a fraction of the required computing time. Factor analysis resulted in very similar correlations of EBV and rank lists for all indices, and equal number of genotypes overlapping with the reference rank lists as obtained with the PCA method, with 28, 24 and 28 genotypes selected with all methods for quality, production and disease index respectively (Fig. 5 bottom panels, Fig. 7b–d).

To visualize the portability and flexibility of the approach, we tested a variety of available software implementations (Table 3). Thus, depending on the situation and requirement of the analysis and data, an analyst can choose among a large smorgasbord of alternatives.

**Table 3.** Available linear mixed-effect model (LMM) implementations and their performance on the first three PCs in the Loblolly pine case study.

Name	Reference	Software platform	Method	Inference algorithm	${\hat{h}}_{PC1}^{2}$	${\hat{h}}_{PC2}^{2}$	${\hat{h}}_{PC3}^{2}$	Computing time [s]	Added info
ASReml-R	Butler *et al*. (2023)	R	Frequentist	AI-REML	0.645 (0.226)	0.170 (0.065)	0.579 (0.198)	0.116 (0.082)	Uses Fortran subroutines
JWAS	Cheng *et al*. (2018)	Julia	Bayesian	Gibbs sampler	0.595 (0.072)	0.192 (0.055)	0.618 (0.101)	5.86 (0.401)	1,000 MCMC iterations
brms	Bürkner (2017) and Carpenter *et al*. (2017)	R	Bayesian	Hamiltonian Monte Carlo sampler	0.706 (0.105)	0.194 (0.071)	0.673 (0.128)	1,260 (63)	Uses RStan, 2,000 HMC iterations,4 chains
Regress	Clifford and McCullagh (2006)	R	Frequentist	Newton-Raphson	0.686 (0.241)	0.178 (0.070)	0.628 (0.217)	7.70 (0.69)	Converged after 5 and 6 iterations
INLA	Rue *et al*. (2009)	R	Bayesian	Integrated nested Laplace approximation	0.647 (0.123)	0.140 (0.025)	0.646 (0.121)	297 (6)	Default settings
BGLR	Perez and de los Campos (2014)	R	Bayesian	Gibbs sampler	0.643 (0.145)	0.273 (0.095)	0.637 (0.154)	11.3 (0.9)	Semi-parametric approach, 1,500 MCMC itertions
sommer	Covarrubias-Pazaran (2016)	R	Frequentist	Direct-Inversion Newton–Raphson or Average Information	0.667 (0.107)	0.178 (0.063)	0.617 (0.109)	3.41 (0.23)	Developed for analysis of dense covariance matrices
rrBLUP	Endelman (2011)	R	Frequentist	REML	0.687 (0.067)	0.178 (0.052)	0.628 (0.071)	7.67 (0.80)	Std dev estimated using subsampling

Computing time is averaged across ten runs with standard deviations within parenthesis. All estimated heritabilities for each PC are in the narrow-sense.

## Discussion

Multitrait LMM analysis to estimate heritabilities, genetic correlations and breeding values is the cornerstone of breeding programs for improving yield, disease resistance and quality in animals, crops, and forest trees. Unfortunately, if many traits are considered jointly, this analysis is far from straight forward to perform due to several reasons, including problems with convergence to a stable solution, required computational time, and precision in parameter estimates. To overcome this hurdle, we propose the use of PCA to reduce the dimension of the phenotypic response variables. We show the benefit of the approach on two data set of Loblolly and Scots pine pedigrees, with a large number of traits recorded at multiple time points. The PCA separated trait groups and REML analysis resulted in a 1,000-fold reduction in computational time as compared to traditional multitrait analysis. Because obtained PCs are orthogonal (to each other), the need to use multivariate analysis is diminished. The individual univariate REML analyses converged after 10 iterations. Rank lists based on estimated breeding values (EBV) obtained from traditional and PCA approaches correlated strongly among the different selection indices used (i.e. production, quality and disease resistance).

In breeding applications, it is not uncommon that the breeding objective traits cannot be measured directly, for example land economic value per hectare at the age of harvest in forest tree breeding programs (Burdon and Klápště 2019). In such cases, several traits are measured that hopefully correlate well with the breeding objective traits. This is typical in breeding of species with long generation turnover, such as forest trees or some livestock animals. In these situations it could be more profitable to rather consider phenotypic profiles than individual traits with unclear connection to future breeding objective traits as diminishing age—age correlations reduces the response to selection (Jansson *et al*. 2003; Dong *et al*. 2019; Lee *et al*. 2024). As some traits are very expensive to measure, such as destructive sampling like meat quality traits in beef cattle (Warner *et al*. 2010), physiological traits in woody plants including fire-induced irreversible xylem damage and low temperature-induced tissue freezing (Li *et al*. 2023), and wood (sawn timber) quality traits (Fukatsu *et al*. 2015; Fundova *et al*. 2020), phenotype profiles could be measured and analysed with PCA to incorporate different types of traits jointly.

In Swedish forest tree breeding programs, there are currently multiple traits in assessment schemes including measurements on tree growth, adaptation and external wood quality. Similar characteristics have been incorporated into other tree breeding programs such as the fourth round of selection in the Loblolly pine breeding program in southeastern USA (Isik and McKeand 2019) and Douglas-fir breeding program in New Zeeland (Dungey *et al*. 2012). It is, however, expected that in the future the number of traits in selection will increase to further mitigate the effects of climate change on forest tree resilience and to aim for more adapted trees. Adaptation traits can be such as resistance to diseases and different pests (Brar *et al*. 2015; Hall *et al*. 2024), spring frost tolerance (Lundströmer *et al*. 2020), drought tolerance (Hayatgheibi *et al*. 2021) and fecundity (Li *et al*. 2023). Furthermore, considering internal wood quality in terms of wood density measurement as a selection trait is under research development and is expected to have greater impact on breeding objectives in the future. Several studies have shown unfavorable genetic correlation between tree growth and wood density which should in that case take into account in breeding to maintain acceptable level of this trait for production purposes (Fundova *et al*. 2018, 2020). Hence, the use of PCA-based trait evaluations could drastically improve efficiency of multiple trait analysis, as both the PCA itself and the following univariate analyses can be conducted with great reduction in computing time without the loss of phenotypic and genetic variation.

In large-scale breeding evaluation systems, such as those provided by Interbull in dairy cattle (https://interbull.org/index), Treeplan in forest trees (http://www.treebreeding.com/technology/treeplan) and INGER in Rice (https://www.irri.org/inger), phenotyping and genotyping efforts are gathered and standardized on a wide geographical scale to perform selections for future generations of breeding. In such large-scale programs, the genetic evaluation system play a crucial role in assessing the genetic merit of individuals. Data collected in trials with crops or forest trees typically need to be standardized, where site-specific effects are removed from the phenotypic records, and genetic parameters must be collected at a population level to enhance nationwide or global comparison between available material. Then, depending on the breeding goal and target zone, all available trait data needs to be weighted together in the final BLUP analysis step. Thus, a number of analysis steps are conducted sequentially, to be able to evaluate all traits accordingly with reasonable accuracy and computing time. However, combining results from multiple PCA of different datasets is not straightforward because PCA is sensitive to the variance structure of the data it is applied to, and each analysis will reflect the unique variance structure (of that particular dataset). Harmonization of the data sets can circumvent this hurdle. For example, preadjustment techniques that remove within site variation, and defining common trait classes that should be included. In addition, incremental PCA (IPCA) (Weng et al. 2003) can be used as a feasible option to merge harmonized datasets into one very large. An alternative is to perform a meta-analysis of the results of each individual PCA to score which traits that are important for respective PC and identify common trends and ranklist similarities. Kim *et al*. (2018) developed a sparse PCA alternative (MetaPCA) by combining the L1-regularization approach of Zou *et al*. (2006) with a penalized matrix decomposition calculation, and showed improved accuracy in analysis of multiple omics datasets in yeast, prostate cancer, mouse metabolism and TCGA pan-cancer methylation. Further effort into this direction is needed to improve large-scale genetic evaluations using PCA-based methods.

Here, we used standard SVD-based PCA and BPCA to obtain orthogonal PCs of all phenotypic traits. There are many alternative directions to improve this dimension reduction step, depending on the characteristics of the phenotypic data and the goal of the genetic evaluations. For example, each obtained PC in these example cases were a mixture of all included traits, albeit some to a very low degree. It is tempting simply to truncate small contributions of some variables, but Cadima and Jolliffe (1995) show that this ad-hoc solution can indeed result in erroneous approximations and poor interpretations. Zou *et al*. (2006) introduced sparse PCA (SPCA) by performing a L1-regularization step via elastic nets so that sparse loadings is obtained, which greatly increases interpretability of the analysis (but see also Jolliffe *et al*. 2003; Gao 2008). In a similar effort, Cox and Arnold (2018) showed how to use simple or Hausman components to improve the interpretability of the analysis which satisfied the Thurstonian criteria (i.e. each component does not containing too many variables and each variable does not being incorporated into many components). These efforts could help in the genetic evaluations of breeding populations when creating selection indices for a more transparent use of PCs.

Although the obtained PCs are orthogonal at the phenotypic scale, this is not necessarily the case for genetic and environmental terms. This discrepancy suggests that analyzing all PCs with univariate linear mixed models (LMMs) may not always be optimal. We observed few indications of nonzero genetic correlations among the PCs in the two examples. Our approach sometimes approximates the actual multivariate model, and other times it provides an equally good alternative. This indicates that further studies are needed to determine under which conditions this univariate approximation is sufficient and what factors influence the level of correlations among PCs. Potential differences in correlations of PCs might be due to several factors. First, if the available traits are not too highly phenotypically correlated and spans all the PC space effectively and evenly (i.e. the PCA loadings), it might help to improve the efficiency of the method as PCA is not restricted when producing the rotation (transformation) of the trait data. Additionally, the sample sizes of the data sets could also be an important factor (Jollife and Cadima 2016; De Marco and Nóbrega 2018), as well as the genetic relationships between the pedigree members (Kerr *et al*. 2015; Momen and Morota 2018; Zhang *et al*. 2018). A larger sample size with a higher proportion of related individuals can help detect a nonzero correlation if it exists, despite limitations in both the PCA and the subsequent LMM analysis. Further studies involving simulations using PCA with univariate LMMs are warranted to determine when and why the univariate approximation is a feasible choice.

In the Scots pine example presented here, all traits were preadjusted prior to the ordination analysis to remove site-specific effects (Calleja-Rodriguez *et al*. 2019), turning all the data as continuous traits, even though some were originally integer counts, such as the number of top shoots of the tree. Similarly, in the Loblolly pine example, all trait data (i.e. estimated breeding values) were adjusted or deregressed following the approach suggested by Garrick *et al*. (2009). Continuous data works very well with PCA, as it relies on linear transformation that identifies the PC maximizing the variance in the data, regardless of the underlying distribution. However, the interpretation of the components is enhanced if the data are normally distributed. Nonnormal data, especially if it includes outliers or is heavily skewed, can affect the estimation of the correlation matrix, which standard PCA relies on (Jolliffe 2002). An alternative is robust PCA methods that are designed to handle data with outliers or noise that traditional PCA might not handle well: the method decomposes the data into a low-rank matrix and a sparse matrix, which can capture corrupted observations (Gao 2008; Wright *et al*. 2009). In addition, alternatives exists for noncontinuous data, such as the multiple correspondence analysis (MCA), which is used for analyzing multivariate data sets containing categorical variables by creating an indicator matrix (a Burt table) from the original variables (Mori *et al*. 2016). To summarize, there exists a smorgasbord of alternative PCA related approaches which can be used in situations of nonnormal noncontinuous data and to improve interpretability of PCA.

While PCA is best suited for continuous data, it is sometimes applied to discrete data in genetic analysis due to its popularity and ease of use. Widely used examples are applying PCA on binary marker data, such as SNPs or insertion-deletion (Indel) markers, to infer ancestral population assignments of the analyzed population or to correct for population stratification in genome wide association studies (GWAS), even though the discrete nature of the marker data violates the assumptions of the PCA. Some alternatives for overcoming this hurdle involves using correspondance analysis (i.e. MCA) or applying model-based alternatives which can handle discrete data in analysis of genetic variation (Bishop 2006; Agrawal *et al*. 2020): further research into this direction is warranted.

In summary, we have shown that PCA can be a viable option in multitrait analysis, and in particular if the number of traits measured is large. By reducing the multitrait LMM to univariate alternatives, computing times can be 1,000-fold reduced while capturing all phenotypic variation of the analyzed population. Several alternatives exists for data imputation to complete multitrait records which allows for the use of PCA of phenotypic profiles in real breeding applications as highlighted in both of our case studies. Another advantage of the proposed approach is that there exists many available implementations of PCA and LMM which can be combined according to the specific application at hand. In our case, we tested some of the available LMM implementations (Table 3) that can accommodate various types of response functions, type of predictor sets and dependencies among those. We believe that PCA-based genetic evaluations can be a part of a population genetic analysts toolbox for accurate and fast multitrait analysis where large-scale phenotyping efforts have been performed.

## Supplementary Material

jkae228_Supplementary_Data

## Data Availability

The Scots pine data are provided at https://github.com/jonhar97/Reduced_phenotype_MME. Supplemental material available at G3 online.
