# Supplementary material for: Principal component analysis revisited: fast multitrait genetic evaluations with smooth convergence
Source: G3 (Bethesda). 2024 Oct 21;14(12):jkae228. doi: 10.1093/g3journal/jkae228 (PMC11631533; doi:10.1093/g3journal/jkae228)
Supplement: jkae228_Supplementary_Data [file jkae228_supplementary_data.pdf]

# Principal component analysis revisited: fast multi-trait genetic evaluations with smooth convergence. Supplemental document

JON AHLINDER*,[1], DAVID HALL[1,2], MARI SUONTAMA[1], AND MIKKO J. SILLANPÄÄ[3]

[1] *Skogforsk, Sävar, SE-91821, Sweden.*
[2] *Department of Ecology and Environmental Science, Umeå University, Umeå, SE-90736, Sweden.*
[3] *Research Unit of Mathematical Sciences, Oulu University, Oulu, FI-90014, Finland.*

## 1. SUPPLEMENTARY FIGURES

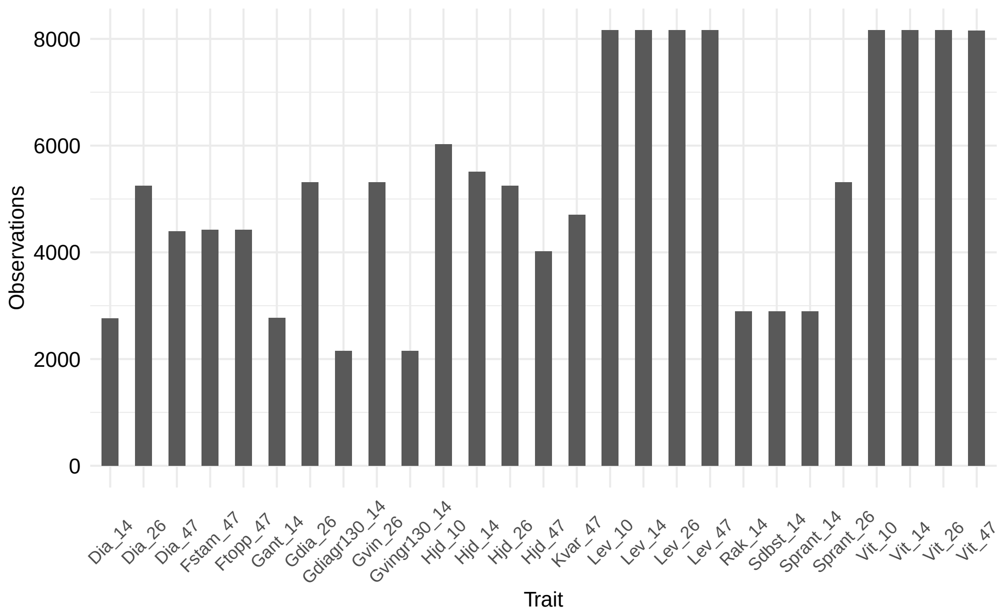


**Fig. S1.** Number of observed phenotypic records for all spatially adjusted traits scored in the Scots pine field trial.

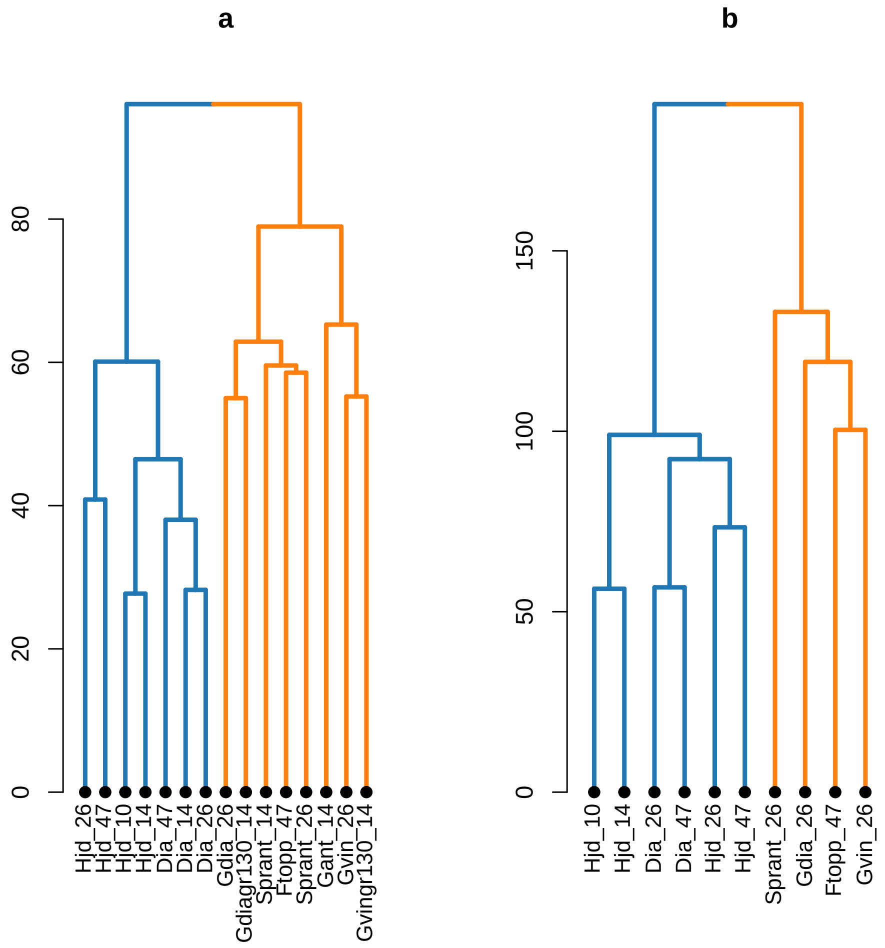


**Fig. S2.** Dendrogram of the Euclidean distance between obtained loadings for: A the 1685 individual Scots pine subset with 15 traits available, and B the 6044 individual subset with 10 traits available. Blue branches highlight production related traits while orange branches highlight tree quality related traits.

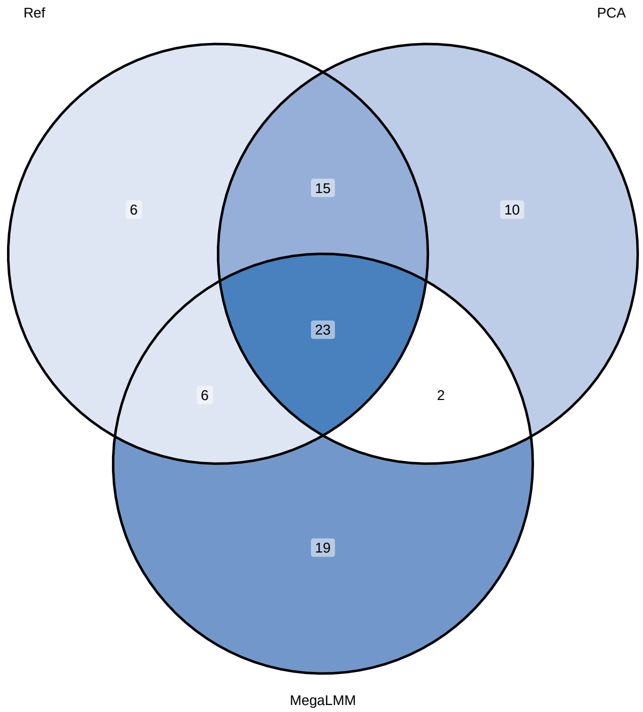


**Fig. S3.** The number of common genotypes among top 50 ranked trees of the quality index obtained by MegaLMM, PCA and full multivariate approaches on the 1685 Scots pine genotypes.

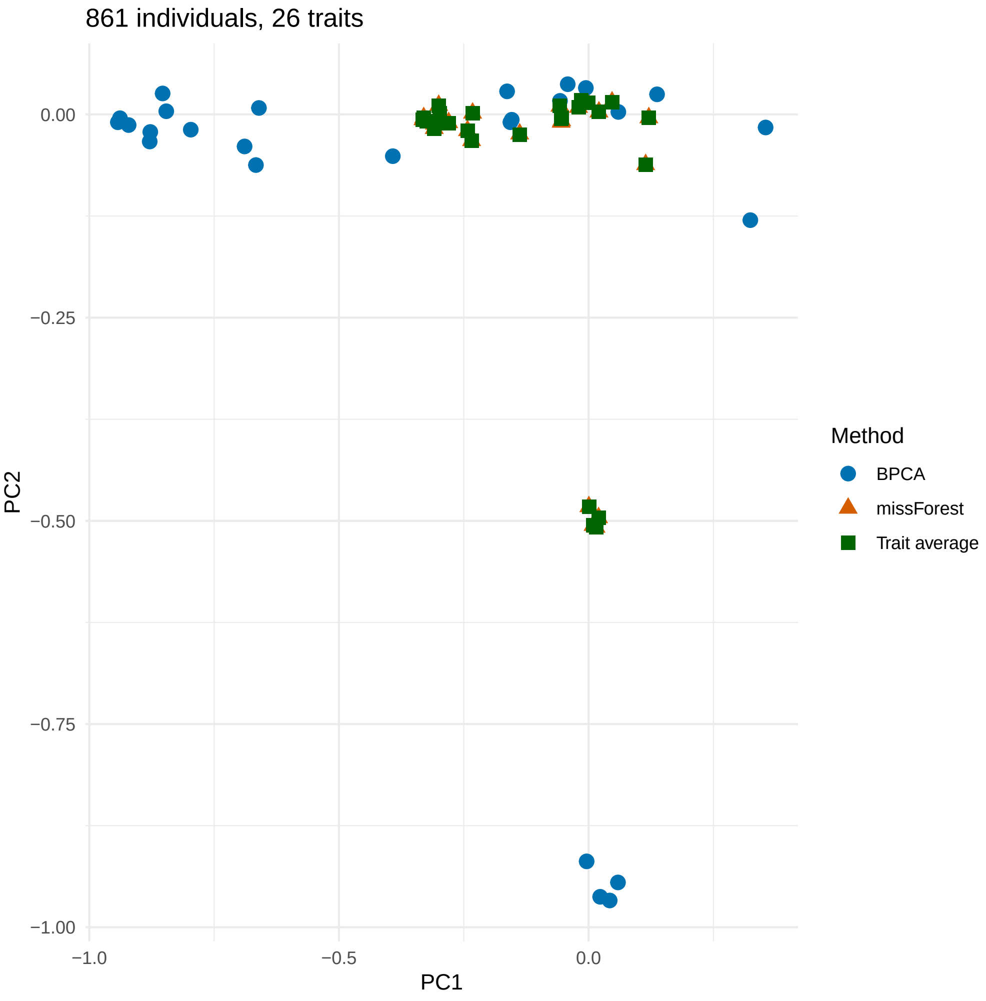


**Fig. S4.** Loading plot of PC1 and PC2 on the Loblolly pine data using the three tested imputation methods BPCA, missForest and trait average.
